# Effect of a recent intradermal test on the specificity of P22 ELISA for the diagnosis of caprine tuberculosis

**DOI:** 10.3389/fvets.2024.1358413

**Published:** 2024-02-08

**Authors:** Carlos Velasco, Javier Ortega, Jaime Ricón, Beatriz Romero, Lucía de Juan, Lucas Domínguez, Mercedes Domínguez, Inmaculada Moreno, Julio Álvarez, Javier Bezos

**Affiliations:** ^1^VISAVET Health Surveillance Centre, Complutense University of Madrid, Madrid, Spain; ^2^Facultad de Veterinaria, Departamento de Sanidad Animal, Universidad Complutense de Madrid, Madrid, Spain; ^3^Unidad de Inmunología Microbiana, Centro Nacional de Microbiología, Instituto de Salud Carlos III, Madrid, Spain

**Keywords:** caprine tuberculosis, intradermal tuberculin test, P22 ELISA, booster effect, specificity

## Abstract

Caprine tuberculosis (TB) is a zoonotic disease caused by members of the *Mycobacterium tuberculosis* complex. TB eradication programs in goats are based on the single and comparative intradermal tuberculin tests (SITT and CITT, respectively). Antibody-based diagnostic techniques have emerged as potential diagnostic tools for TB. P22 ELISA has been previously evaluated using samples collected after the intradermal tuberculin tests to maximize the sensitivity, a phenomenon known as booster effect. However, there is no information available on whether the use of this diagnostic strategy could lead to a decrease of its specificity (Sp). The aim of the present study was to elucidate the interference effect of a recent CITT on the Sp of the P22 ELISA in serum and milk samples collected at different times after the CITT from a TB-free herd (n = 113). The number of reactors to P22 ELISA was significantly higher (*p* < 0.01) on serum samples collected 15 days post-CITT compared to day 0, showing a decrease in Sp from 99.1% (95% CI; 95.2–99.8%) to 88.5% (95% CI; 81.3–93.2%). The number of reactors and the quantitative values of P22 ELISA were significantly higher (*p* < 0.01) in serum samples compared to milk. No significant (*p* > 0.05) changes in the Sp of the P22 ELISA were observed throughout the different time samplings using milk No significant (*p* > 0.05) changes were observed on days 30 and 60 post-CITT. In conclusion, the booster effect strategy may significantly decrease the Sp of P22 ELISA in TB-free herds when serum samples are used but not when milk is tested.

## Introduction

Tuberculosis (TB) in goats, mainly caused by *Mycobacterium bovis* and *M. caprae,* is a chronic infectious disease that affects caprine herds worldwide (1, 2). It poses a threat to animal health, causing economic losses to the livestock industry, and has implications for public health due to its zoonotic potential (3–5). In contrast to TB in cattle, goats are not subjected to compulsory eradication programs within the European Union (EU), although the scope of the bovine TB eradication program also includes caprine herds that are epidemiologically linked to bovine herds (6). Furthermore, some regions of Spain have implemented regional caprine TB eradication programs (7). These programs are based on test and cull strategies using the single and comparative intradermal tuberculin tests (SITT and CITT, respectively) and, in very specific cases, the interferon-gamma release assay (IGRA), all of which are based on cellular immune response detection (8). However, these diagnostic tools have limitations in terms of sensitivity (Se) and specificity (Sp), mainly when animals do not develop an appropriate cellular immune response, e.g., on advanced stages of infection (3, 9). For this reason, in recent years techniques based on antibody (Ab) detection have emerged as ancillary diagnostic tools to detect TB-infected animals, especially those that do not react to cellular tests (10). Moreover, humoral assays have certain advantages such as their high Sp and the variety of sample types supported, including serum (10, 11), milk (12) and even oral fluid (13) and urine samples (14). Furthermore, since they do not have high logistical demands and do not require immunological stimulation with antigens prior conducting the assay, there is interest in its assessment and development (11). However, it is well-known that the Se of these techniques is lower than that reported when using the intradermal tuberculin test and IGRA, especially in recently TB-infected animals, making it difficult to detect the disease in certain epidemiological situations (8). Several studies have reported an increase in serum (10, 15–17) and milk (18, 19) Ab titres after a recent intradermal tuberculin test (booster effect) in ruminants, leading to an improvement of the Se of techniques based on their detection (15, 20, 21). In this context, P22 ELISA is an immunoassay that detects specific Ab against a multiprotein antigen, which is affinity-purified from bovine purified protein derivative (PPD) (21). This tool has been previously evaluated in cattle (10) and goats (12, 19) subjected to a previous intradermal test, leading to an increase in the number of reactors to P22 ELISA when using serum and milk samples. This suggests the potential of the booster effect for maximizing Se in TB-infected herds (19). However, to the best of our knowledge, no studies have been carried out in caprine TB-free herds in order to evaluate the effect of this diagnostic strategy on the Sp of P22 ELISA or other humoral diagnostic test. Therefore, in order to obtain a better knowledge of this diagnostic strategy, the objective of the present study was to evaluate the Sp of the P22 ELISA using serum and milk samples collected at different times (0, 15, 30, and 60 days) after a CITT in a TB-free caprine herd.

## Method

### Study design

**Figure 1:**
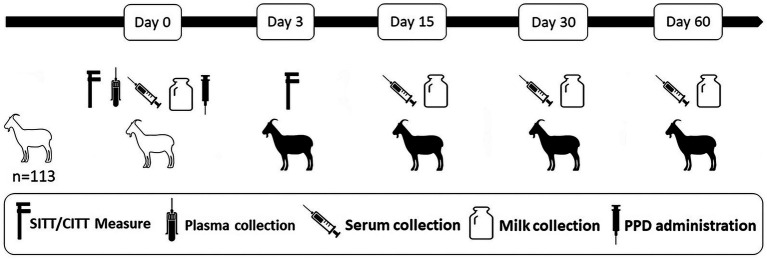
Summary of the experimental design. Goats were subjected to single intradermal tuberculin test (SITT), comparative intradermal tuberculin test (CITT) and interferon-gamma release assay (IGRA) at day 0 of the study and tested using P22 ELISA in serum and milk samples at days 0, 15, 30, and 60 after the intradermal test. White and black silhouettes represent goats before and after the PPD administration, respectively.

The study was carried out in a dairy herd of Murciano-Granadina breed goats located in the central region of Spain. The herd (*n* = 113) was negative in the annual tests performed within the framework of a regional eradication program in the last 7 years and had no previous history of TB. Animals in the herd were subjected to SITT, CITT and IGRA at the beginning of the study (day 0). All animals were also tested using P22 ELISA in serum and milk samples at days 0, 15, 30 and 60 after the intradermal test. Milk and blood samples from day 0 were obtained previously to the PPDs inoculation (Figure 1).

### Indirect ELISA

Individual milk (40 mL) and blood (10 mL) samples were collected in plastic tubes during the milking session from the mammary gland and jugular vein, respectively. In the laboratory, both types of samples were processed as described previously (19). Briefly, an aliquot of 1 mL of each milk sample was centrifuged at 13,000 g for 5 min, after which the superficial cream and pellet were discarded. Blood samples were centrifuged at 1,500 g for 10 min in order to obtain the serum. Milk and serum samples were stored at −20°C until performing the ELISA assay. Then, serum and milk samples were analysed by employing an in-house indirect ELISA for detection of specific Ab against the P22 protein complex (P22 ELISA) (22). The P22 ELISA was performed as described previously using the optimal dilution of serum (1/100) and milk (1/8) samples in 5% skimmed milk/PBS solution (12, 19). Finally, the optical density (OD) was measured at 492 nm with an ELISA reader. The serum and milk sample results were expressed as an ELISA percentage (E%), calculated based on the following formula: sample E% = [mean sample OD / (2 x mean of negative control OD)] x 100. Serum and milk samples with E% values greater than 100E% or 150E% were considered reactor to P22 ELISA when applying the standard and severe criteria, respectively, as described previously (23, 24).

### Intradermal tuberculin tests

SITT and CITT were performed according to the protocol published by the European Union Reference Laboratory (EU-RL) for bovine TB (SOP/002/EURL), Regulation EU 2016/429, Commission Delegated Regulation EU 2020/688 and Spanish Royal Decree 2611/1996. Briefly, the SITT consisted in the intradermal inoculation of 0.1 mL of bovine PPD (CZ Vaccines, Porriño Spain) on the left-hand side of the neck by using a Dermojet syringe (Akra Dermojet, France). For the CITT, an intradermal inoculation of 0.1 mL of avian PPDs on the right side of the neck was also performed. The results were interpreted as described previously (24). Two cut off points were applied in this study (Table 1): to read the results of the SITT according to the standard interpretation, an increase in skin fold thickness ≥ 4 mm and/or the presence of clinical signs (oedema, pain, exudation or necrosis) were considered a positive reaction to the SITT. When using the severe interpretation, a skin-fold thickness increase of >2 mm and/or the presence of clinical signs were considered positive. For the standard interpretation of the CITT, an animal was considered positive if the bovine reaction was >4 mm greater than the avian reaction and/or clinical signs on the bovine PPD inoculation site were observed. When using the severe interpretation, reactors were those with a bovine reaction >2 mm greater than the avian reaction and/or with clinical signs on the bovine PPD inoculation site.

**Table 1:** Number of negative animals (N), and percentage of specificity (Sp, including Wilson’s 95% CI) to the different diagnostic tests used in the present study.

Technique	Criteria/ cut-off point	Day 0		Day 15		Day 30		Day 60	
		N	Sp (95% CI)	N	Sp (95% CI)	N	Sp (95% CI)	N	Sp (95% CI)
SITT^a^	Standard	113	100% (96.7–100%)	–	–	–	–	–	–
	Severe	113	100% (96.7–100%)	–	–	–	–	–	–
CITT^b^	Standard	113	100% (96.7–100%)	–	–	–	–	–	–
	Severe	113	100% (96.7–100%)	–	–	–	–	–	–
IGRA^c^	0.05	113	100% (96.7–100%)	–	–	–	–	–	–
	0.1	113	100% (96.7–100%)	–	–	–	–	–	–
P22 ELISA^d^ serum	100	112	99.1% (95.2–99.8%)	100	88.5% (81.3–93.2%)	107	94.7% (88.9–97.5%)	108	95.6% (90.0–98.1%)
	150	113	100% (96.7–100%)	103	91.1% (84.4–95.1%)	109	96.4% (91.2–98.6%)	109	96.4% (91.2–98.6%)
P22 ELISA^d^ milk	100	112	99.1% (95.2–99.8%)	113	100% (96.7–100%)	108	95.6% (90.0–98.1%)	111	98.2% (93.8–99.5%)
	150	112	99.1% (95.2–99.8%)	113	100% (96.7–100%)	110	97.3% (92.4–99.1%)	112	99.1% (95.2–99.8%)

^a^An animal was considered a positive reactor to the single intradermal tuberculin test (SITT) when there was an increase of ≥4 mm (standard interpretation) or > 2 mm (severe interpretation) in the skin fold thickness and/or the presence of clinical signs (oedema, pain, exudation or necrosis) was observed.

^b^An animal was considered a positive reactor to the comparative intradermal tuberculin test (CITT) when the bovine reaction was greater than the avian reaction by more than 4 mm (standard interpretation) or the bovine reaction was ≥3 mm and greater than the avian reaction (severe interpretation); and/or there were clinical signs (oedema, pain, exudation or necrosis) at the bovine purified protein derivative (PPD) inoculation site.

^c^An animal was considered positive to the interferon-gamma release assay (IGRA) if the optical density (OD) of a sample stimulated with bovine PPD minus the OD of PBS was greater than 0.1 (standard interpretation) or 0.05 (severe interpretation) and greater than the OD of the sample stimulated with avian PPD.

^d^An animal was considered positive to P22 ELISA when the E% value was greater than 150 (standard interpretation) or 100 (severe interpretation).

### Interferon-gamma release assay (IGRA)

Blood samples were collected before the skin test (day 0 of the study) from the jugular vein by venepuncture using evacuated tubes (BD Vacutainer Becton, Dickinson and Company, Franklin Lakes, United States) with lithium heparin (Figure 1). In the laboratory, blood samples were stimulated with bovine and avian PPDs (CZ Vaccines, Porriño, Spain) at a final concentration of 20 μg/mL in order to evaluate the IFN-γ production. Afterwards, the blood samples were processed as described elsewhere (25). IFN-γ production in plasma was measured using a commercial IGRA (Bovigam TB kit, Thermo Fisher Scientific, Waltham, United States) according to the manufacturer’s instructions. An animal was considered positive to the IGRA if the optical density (OD) of a sample stimulated with bovine PPD minus the OD of PBS was greater than 0.1 (standard interpretation) or 0.05 (severe interpretation) and greater than the OD of the sample stimulated with avian PPD (26).

### Statistical analysis

Data were analysed using the SPSS Statistics 25 commercial statistical software (IBM, New York, NY, United States), and interpreted by considering a value of *p* of 0.05 in order to determine statistical significance. Wilson’s 95% confidence intervals (95% CI) were calculated for the percentage of animals that were positive to the different tests using WinPepi version 11.6 (27). The comparison of the proportions of P22 ELISA reactors using milk and serum samples on the different sampling events was performed using Cochran’s Q test. Quantitative differences in the E% during the study concerning the booster effect were analysed using the Friedman test.

## Results

**Figure 2:**
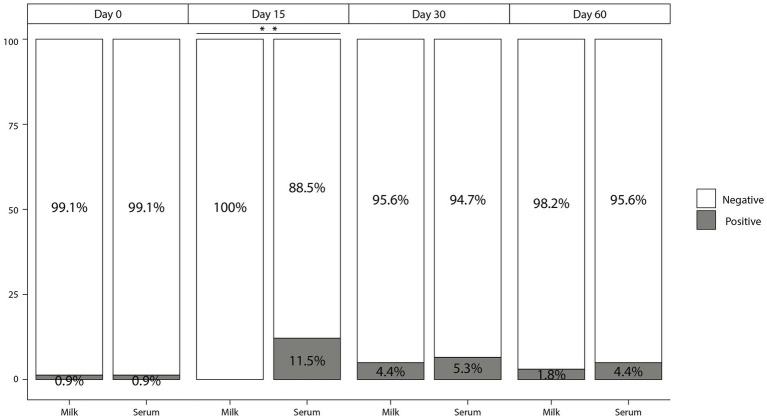
Percentage of animals that tested positive (grey) and negative (white) to P22 ELISA in milk and serum samples with a cut-off point of 100 E% out of the 113 animals analysed during the different testing events of the period evaluated. **p < 0.01.

**Figure 3:**
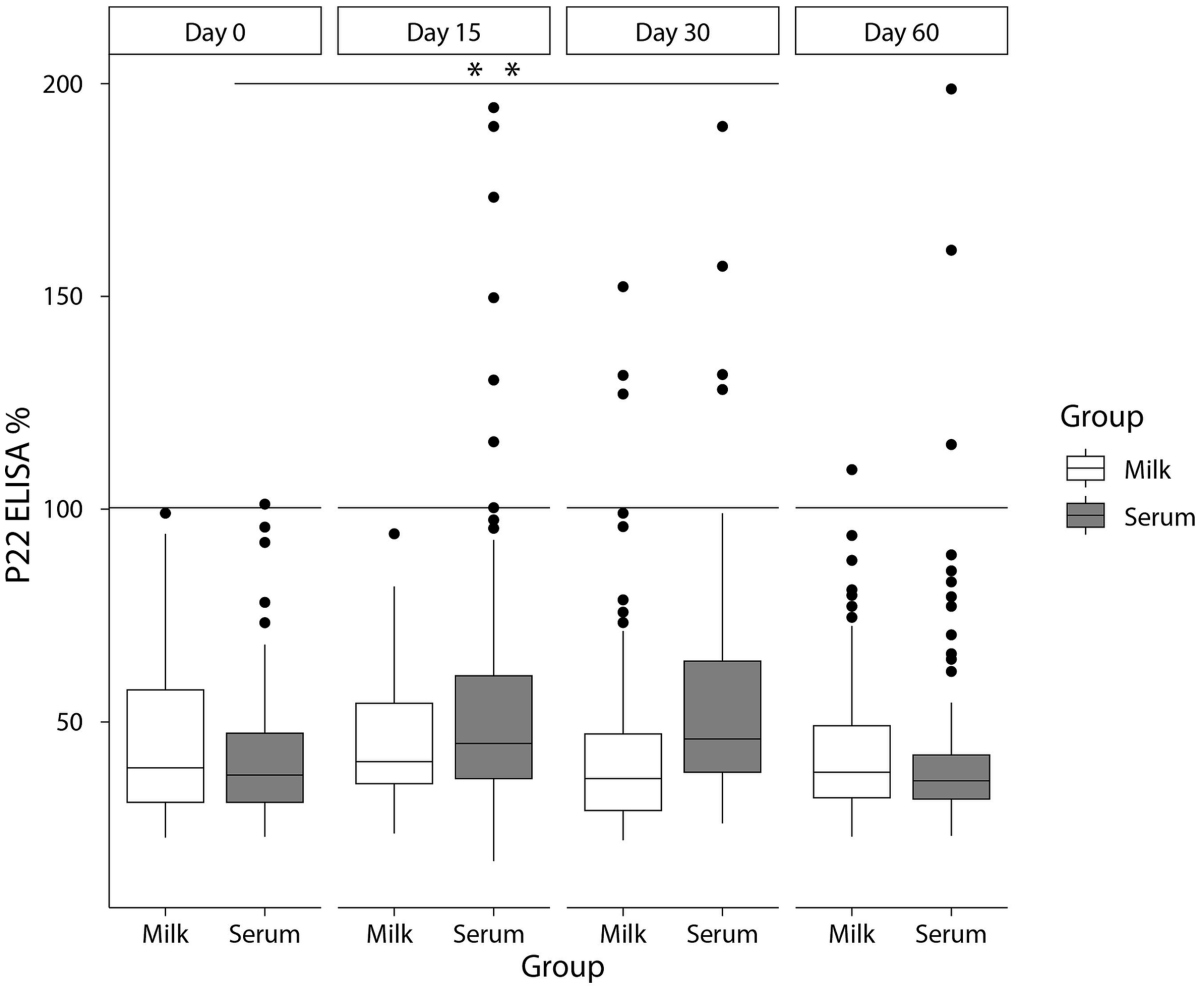
E% values in milk (white) and serum (grey) at days 0, 15, 30, and 60 of boosting effect study in TB-infected goats with a cut-off point of 100 E%. **p < 0.01.

The number and percentage of animals testing negative to the different diagnostic tests are summarized in Table 1. No reactors to the SITT, CITT or IGRA were detected at day 0 of the study regardless of the test interpretation (standard or severe) used (Table 1). Regarding P22 ELISA results in serum samples in the initial day of the study (day 0), no reactors were observed when using the standard interpretation [100% Sp (95% CI; 96.7–100%)] (Table 1; Figure 2) and only one goat was positive to P22 ELISA when using the severe interpretation [99.1% Sp (95% CI; 95.2–99.8%)]. Afterwards, both the quantitative response (E%) and the number of reactors in the P22 ELISA using serum samples collected on day 15 increased significantly (*p* < 0.01) compared to day 0 when using the standard [91.1% Sp (95% CI; 84.4–95.1%)] and severe interpretation [88.5% Sp (95% CI; 81.3–93.2%)]. However, only significant differences (*p* < 0.05) in the quantitative response were observed on day 30 when using serum samples (Figure 3).

Regarding milk samples, only one animal was reactor to P22 ELISA [99.1% Sp (95% CI; 95.2–99.8%)] when using milk samples collected on day 0 and regardless of the criteria applied. No significant differences (*p* > 0.05) were observed in E% values and percentage of reactors in the P22 ELISA when using samples collected 15 days after the PPD inoculation, yielding a Sp of 100% (95% CI; 96.7–100%) of the P22 ELISA at this time. Significant (*p* < 0.01) differences on both E% values and number of reactors (regardless of the criteria applied) were observed in serum samples compared to milk on day 15 (Figures 2, 3). In following sampling events a decrease in the E% values were observed when using milk (or serum) samples up to day 60 of the study, with levels similar to those reported on day 0 of the study (Figure 3). The Sp of P22 ELISA on days 30 and 60 of the study was higher, when using milk compared to serum samples, although differences were not significant (Table 1; Figure 2).

## Discussion

In the present study, we aimed to elucidate the impact of a recent CITT on the Sp of P22 ELISA in serum and milk samples collected at several times from a TB-free caprine herd. In this study, we demonstrated a significantly lower Sp of P22 when serum samples were collected 15 days after the PPDs administration compared to day 0. However, no significant changes in the Sp of the test after the skin test were observed when milk samples were used regardless of the time of sampling.

The increase in the specific Ab against MTBC members production after a recent intradermal test (booster effect) has been widely characterized and suggested as a suitable methodology to increase diagnostic Se in a variety of animal species, including TB-infected cattle (10, 16), goats (11, 19, 21), new world camelids (20) and red deer (28). However, the increase of reactors as a consequence of a previous intradermal test could also potentially lead to a significant reduction in the Sp of Ab-based diagnostic tests, as observed by the results in the present study.

In agreement with the timeline followed in a recent study using the booster effect in TB-infected goats (19), the present study evaluated the performance of the P22 ELISA on serum and milk samples taken from a TB-free caprine herd on the 15, 30 and 60 days following a CITT. Our results showed a significant increase of the quantitative values (E%) of the P22 ELISA in serum samples collected 15 days after the PPDs administration, which led to a significantly higher number of reactors (regardless the criteria applied) to the P22 ELISA at day 15 compared to day 0. Similar results were reported in previous studies that used the booster effect to increase the Se of humoral test in TB-infected cattle and caprine herds when using serum (15, 21) or milk samples (18, 19). In fact, a previous study in TB-infected cattle also reported an increase of the Ab-based immune response when milk samples were collected after the performance of the intradermal tuberculin test (18). In TB-infected goats, two previous studies reported similar results using serum samples collected 15 days after the performance of the intradermal tuberculin test (11, 21). Gutiérrez and collaborators (21) observed an increase of Se of a humoral Ab-detection technique from 54.9% to 88.6% when comparing results obtained before and 15 days after the inoculation of PPDs. Similarly, Bezos and collaborators (11) observed an increase in the Se of an experimental P22 ELISA before/15 days after a skin test from 83.2% to 100%. Moreover, the results of a recent study suggested that the booster effect observed on milk or serum samples collected post-skin test could maximize the detection of TB-infected goats in dairy herds (19). In this study the E% values of animals from an infected herd were significantly higher 15 days after the intradermal test than those observed on days 30 and 60 of the study, what suggests a better performance of the P22 ELISA when using serum or milk samples collected at this sampling event and detecting the 100% of TB-infected goats (19). The booster effect has also been assessed for the humoral diagnosis of paratuberculosis (PTB), another mycobacterial disease affecting domestic ruminants caused by *Mycobacterium avium* subsp. *paratuberculosis* (MAP) (29, 30). In this regard, a recent CITT may maximize the Se of PTB humoral techniques in PTB-infected herd (30), although an impact on the Sp observed PTB-free herds has been also described (29).

Since the totality of studies performed in ruminants (cattle or goats) in order to evaluate the booster effect in TB humoral response have been carried out in TB-infected herds (11, 15, 18, 19, 21), it is essential to evaluate the effect in TB-free herds to assess the suitability of this diagnostic strategy when the disease is not present. In this context, the present study is the first to evaluate the effect of a recent CITT on the Sp of the P22 ELISA in serum and milk samples collected from a TB-free caprine herd.

In conclusion, the increase in the E% levels after a PPD inoculation led here to a significant increase in the number of (false) positive animals to the P22 ELISA when using serum samples collected 15 days after the PPDs administration, suggesting the Sp of the test could be compromised under these circumstances. However, CITT do not significantly interfere the Sp of P22 ELISA in serum samples collected 30 days post-CITT onwards. In contrast, the collection of milk samples 15 days after a CITT could be a valuable TB diagnostic strategy in caprine herds in combination with ELISA tests since previous results demonstrated a high Sp and a significant increase in the Se, although being the latter lower than that reported using serum samples. The results of the present study will be valuable for the strategic use of Ab-based platforms for caprine TB diagnosis as an ancillary diagnostic tool within the framework of the different caprine TB eradication programs.

## Data availability statement

The original contributions presented in the study are included in the article, further inquiries can be directed to the corresponding author.

## Ethics statement

The animals included in this study were not experimental animals. All samplings and other procedures were carried out in accordance with Spanish legislation (Royal Decree 2611/1996).

## Author contributions

CV: Writing – review & editing, Formal analysis, Writing – original draft. JO: Formal analysis, Writing – original draft, Writing – review & editing. JR: Data curation, Writing – review & editing. BR: Writing – review & editing, Resources, Data curation. LJ: Writing – review & editing, Investigation, Supervision. LD: Writing – review & editing, Conceptualization, Resources. MD: Writing – review & editing. JÁ: Writing – review & editing. JB: Writing – review & editing, Formal analysis, Data curation.
